# Correlational analysis and predictive validity of psychological constructs related with pain in fibromyalgia

**DOI:** 10.1186/1471-2474-12-4

**Published:** 2011-01-08

**Authors:** Sara Maurel, Baltasar Rodero, Yolanda Lopez-del-Hoyo, Juan V Luciano, Eva Andrés, Miquel Roca, Raquel del Moral Bergos, Yolanda Ruiz-Lancina, Javier García-Campayo

**Affiliations:** 1 Servicio de Psiquiatría, Hospital Miguel Servet y Universidad de Zaragoza, Spain; 2 Centro Rodero. Clínica de Neurociencias, Santander, Spain; 3 Departamento de Psicología. Universidad de Zaragoza, Spain; 4 Parc Sanitari Sant Joan de Déu & Fundació Sant Joan de Déu. Sant Boi de Llobregat, Barcelona, Spain; 5 CIBER Epidemiología y Salud Pública, Unidad Epidemiología Clínica, Hospital 12 de Octubre, Madrid, Spain; 6 Institut Universitari d'Investigació en Ciències de la Salut, Hospital Juan March, Universitat de les Illes Balears, Palma de Mallorca, Baleares, Spain; 7 Grupo de Bioinformación. Instituto Aragonés de Ciencias de la Salud, Zaragoza, Spain; 8 Psicóloga. Asociación Aragonesa de Fibromialgia y Fatiga Crónica, Zaragoza, Spain; 9 Grupo Aragonés de Investigación en Atención Primaria, Red de Actividades Preventivas y de Promoción de la Salud (REDIAPP) (G06/170). Instituto Aragonés de Ciencias de la Salud (IACS), Aragon, Spain

## Abstract

**Background:**

Fibromyalgia (FM) is a prevalent and disabling disorder characterized by a history of widespread pain for at least three months. Pain is considered a complex experience in which affective and cognitive aspects are crucial for prognosis. The aim of this study is to assess the importance of pain-related psychological constructs on function and pain in patients with FM.

**Methods:**

**Design**

Multicentric, naturalistic, one-year follow-up study.

*Setting and study sample*. Patients will be recruited from primary care health centres in the region of Aragon, Spain. Patients considered for inclusion are those aged 18-65 years, able to understand Spanish, who fulfil criteria for primary FM according to the American College of Rheumatology, with no previous psychological treatment.

**Measurements**

The variables measured will be the following: main variables (pain assessed with a visual analogue scale and with sphygmomanometer and general function assessed with Fibromyalgia Impact Questionnaire, and), psychological constructs (pain catastrophizing, pain acceptance, mental defeat, psychological inflexibility, perceived injustice, mindfulness, and positive and negative affect), and secondary variables (sociodemographic variables, anxiety and depression assessed with Hospital Anxiety and Depression Scale, and psychiatric interview assessed with MINI). Assessments will be carried at baseline and at one-year follow-up.

**Main outcome**

Pain Visual Analogue Scale.

**Analysis**

The existence of differences in socio-demographic, main outcome and other variables regarding pain-related psychological constructs will be analysed using Chi Square test for qualitative variables, or Student *t *test or variance analysis, respectively, for variables fulfilling the normality hypothesis. To assess the predictive value of pain-related psychological construct on main outcome variables at one-year follow-up, use will be made of a logistic regression analysis adjusted for socio-demographic and clinical variables. A Spearman Rho non-parametric correlation matrix will be developed to determine possible overlapping between pain-related psychological constructs.

**Discussion:**

In recent years, the relevance of cognitive and affective aspects for the treatment of chronic pain, not only in FM but also in other chronic pain diseases, has been widely acknowledged. However, the relative importance of these psychological constructs, the relationship and possible overlapping between them, or the exact meaning of them in pain are not enough known.

## Background

Fibromyalgia (FM) is a prevalent and disabling disorder characterized by a history of widespread pain for at least three months and patient reporting of tenderness in at least 11 out of 18 defined tender points when digitally palpated with about 4 kg per unit area of force [1]. In addition to pain, the patient suffers from other symptoms such as non-restorative sleep, paraesthesia, difficulties in concentration and memory, stiffness, depression and anxiety [2]. FM is a common disorder; its prevalence within the community has been established in the range of 2-3%, predominantly in women in their third to fifth decade [3]. The aetiology of FM is uncertain but the existence of central sensitization is widely accepted [4]. Many treatments have been used in these patients but their efficacy is rather limited [5]. Due to its high prevalence, severe disability, low quality of life for the patient and significant health costs, FM is considered a public health problem [6,7].

Pain is the most common and disabling symptom in FM. The neurobiology of the pain process in FM using neuroimage techniques has developed greatly in recent years, identifying what has been called "pain matrix", which includes the primary and secondary sensory-motor cortex, insula, anterior cingulate, thalamus, dorso-lateral prefrontal cortex and basal ganglia [8-10].

Pain is considered a subjective and complex experience, in which affective and cognitive aspects are crucial for prognosis. At present, classical approaches focusing on modifying coping strategies [11] or on the cognitive restructuring of pain [12] are being widened to include other treatments based on pain acceptation and other psychological constructs. Some of the most important of these constructs are the following:

- *Pain catastrophizing: *This is the most studied pain-related construct. It has been described as an exaggerated negative orientation towards pain stimuli. It is made up of three dimensions: a) rumination: incapacity to avoid thinking about pain; b) magnification: exaggeration of the impact that pain produces; and c) helplessness: the feeling that one cannot free without overcome pain [13]. Some of the consequences that have been associated with pain catastrophizing in patients with chronic pain are more intense pain, heightened pain behaviour, greater consumption of analgesics, reduced involvement in daily activities, occupational disability and suicidal ideation [13,14].

- *Pain Acceptance *[15,16]: This is one of the most promising and valuable pain-related constructs. It comes from contextual or third wave psychotherapies and involves the individual reducing unsuccessful attempts to avoid or control pain and focusing instead on participation in valued activities and the pursuit of personally relevant goals. Some studies have demonstrated that patients with chronic pain and high pain acceptance present less pain, anxiety and depression, and better occupational status. Acceptation is associated with better adaptation to pain independently of pain severity, anxiety or depression.

- *Mental defeat *[17]: This is a recently described cognitive phenomenon not previously assessed in chronic pain, but thoroughly studied in other disorders such as depression and post-traumatic stress disorder. It can be defined as the perceived loss of autonomy in the face of uncontrollable traumatic events, resulting in the person giving up efforts to retain identity and self-will. With regard to pain, mental defeat can be considered a type of self-processing in which repeated episodes of persistent and disabling pain generate negative thoughts on pain and oneself.

- *Pain psychological inflexibility *[18]: From the point of view of third-generation psychotherapies such as acceptation and commitment therapy (ACT), observing external phenomena separately from our thoughts is crucial if we are to act in accordance with our aims and values. The inability to maintain our values in the presence of painful or disabling physical symptoms, emotions and thoughts has been called psychological inflexibility.

- *Perceived injustice *[19]: In situations in which the injury or disease has been produced as a result of negligence or a mistake caused by others (at least from the patient's point of view), the victim experiences his life with a feeling of injustice. The key point in this regard is the feeling of unnecessary suffering that can be blamed on another, requiring reparation from society. There is a clinical perception, which has not been systematically studied, that a feeling of perceived injustice is associated with poor recovery in chronic pain disorders [19].

- *Mindfulness: *This concept is defined as the practice of being mindful, focused on the present moment and without judging the situation we have to live through [20]. It has been demonstrated that mindfulness correlates positively with several measures of psychological well-being and that it is associated with significant long-term improvement in patients with pain and, specifically, in FM [20,21].

- *Positive and negative affect: *Affect has been described as the observable expression of emotion, which can be both positive and negative [22,23]. Negative affect has been related with poor outcomes in FM [22,23], but recently the emphasis has been put on the affect balance style (relative levels of negative and positive affect) as a prognostic marker in FM [24].

It is not currently known whether these psychological constructs correlate between themselves or even overlap each other. In addition, their specific significance for prognosis and treatment on chronic pain disorders has not been assessed. The general aim of this study is to assess the importance of pain-related psychological constructs on function and pain in patients with FM. The specific objective is to assess correlations and possible overlapping among constructs.

## Methods/Design

### Design

Multicentric, naturalistic, one-year follow-up study.

### Setting and study sample

Patients will be recruited from any of the primary care health centres in the region of Aragon, Spain. General practitioners will include patients with FM fulfilling the study criteria until the required sample is completed, without a quota of patients assigned for each centre.

Patients considered for inclusion are those aged 18-65 years, able to understand and read Spanish, who fulfil criteria for primary FM according to the American College of Rheumatology [1], with no previous psychological treatment, and on providing signed informed consent. Those excluded will be patients with severe Axis I psychiatric disorders (dementia, schizophrenia, paranoid disorder, alcohol and/or drug use disorders), severe Axis II disorders or somatic disorders which, from the clinician's point of view, prevent patients from carrying out a psychological assessment.

### Measurements

The variables and instruments of measurement are summarized in Table 1.

**Table 1:** Study variables

Instrument	Assessment area	Time(s) of assessments	Applied by
Sociodemographic data form assistant	Sex, age, marital status, educational level, occupation, income	Baseline	Research

Clinical variables	Family & personal medical & psychiatric history, length of the disorder, main symptoms, psychiatric comorbidity	Baseline	Research

SPPI psychiatric interview [37]	Psychiatric diagnosis	Baseline	Psychiatrist
Pain catastrophizing scale [31]	Severity of catastrophizing	Baseline	Research

Pain acceptance scale [32]	Acceptation of pain	Baseline	Research

Pain Self-Perception Scale (PSPS) [33]	Mental defeat	Baseline	Research

Psychological Inflexibility in Pain Scale (PIPS) [34]	Pain psychological inflexibility	Baseline	Research

Injustice Experience Questionnaire (IEQ) [19]	Perceived injustice	Baseline	Research

Mindful Attention Awareness Scale (MAAS) [21]	Mindfulness	Baseline	Research

Positive & Negative Affect (PANAS) [36]	Positive and Negative Affect	Baseline	Research

Hospital Anxiety Depression Scale (HADS) [40]	Anxiety, depression	Baseline	Research

Fibromyalgia Impact Questionnaire [26]	General function	Baseline and follow-up	Research

Pain Visual Analogue Scale (PVAS) [29]	Pain	Baseline and follow-up	Research

Pain assessed by sphygmomanometer [30]	Pain	Baseline and follow-up	Research

#### Main outcome variables

- Fibromyalgia Impact Questionnaire (FIQ) [25]: The FIQ is a 10-item self-report questionnaire developed to measure the health status of FM patients. The first item focuses on the patient's ability to carry out muscular activities. In the next two items, patients are asked to circle the number of days in the past week they felt good and how often they missed work. Finally, the last seven questions (job ability, pain, fatigue, morning tiredness, stiffness, anxiety and depression) are measured by a visual analogue scale (VAS). The Spanish validated version will be used [26].

- Pain Visual Analogue Scale (PVAS): The PVAS was designed to allow a thorough and understandable subjective assessment of pain. A Visual Analogue Scale is usually a 10-cm horizontal line, with perpendicular lines on the edges, defined as the extreme limits of pain experience. Anchoring points at each edge are characterised by verbal expressions such as "No pain" (accompanied by the number "0") at one end and "maximum pain ever experienced" (accompanied by the number "100") at the other end. The adequate psychometric properties of PVAS have been demonstrated in previous studies [27,28]. The Spanish validated version of this questionnaire will be used [29].

- Pain threshold assessed by sphygmomanometer: the sphygmomanometer, a universally used clinical test, has been demonstrated to be helpful in the identification of patients with FM [30]. It has been recommended that the blood pressure cuff be inflated at an approximate rate of 10 mm Hg per second up to 180 mm Hg or to the point where pain is elicited. Healthy persons usually experience pain when the blood pressure cuff is inflated to 160 mm Hg or more; however, patients with FM usually experience pain at 100-110 mm Hg or at even lower pressure [30].

#### Secondary variables

##### Socio-demographic variables

The following socio-demographic data will be collected: sex, age, marital status (single, married/relationship, separated/divorced, and widowed), educational level (no studies, primary, lower secondary, upper secondary, and university), occupation and income. In addition, relevant clinical variables (family and personal medical and psychiatric history, length of the disorder, main symptoms and medical comorbidity) will be also assessed.

##### Pain-related psychological constructs

- Pain catastrophizing: One of the most used questionnaires for measuring this construct is the Pain Catastrophizing Scale [13]. This is a self-administered 13-item questionnaire that assesses three dimensions: rumination, magnification and helplessness. Each item is scored from 0 (not at all) to 4 (always), and total scores range from 0 to 52. It has good temporal stability, internal consistency and validity. The Spanish validated version will be used [31].

- Pain acceptance [15,16]: This variable is assessed with the Chronic Pain Acceptance Questionnaire [15]. This is 20-item self-administered inventory. There are two principle factors measured by this questionnaire: activities engagement and pain willingness. All items are rated on a 0 (never true) to 6 (always true) scale. The maximum possible total score is 120, with a higher score indicating better acceptance. The Spanish validated version will be used [32].

- Mental defeat [17]: This concept will be evaluated with the Pain Self-Perception Scale. This is a 24-item self-administered questionnaire. These statements are to be rated on a 5-point scale (0 = "Not at all/Never", 1 = "Very little", 2 = "Moderately", 3 = "Strongly", 4 = "Very strongly"), generating a total score ranging from 0 to 96. The Spanish validated version will be used [33].

- Pain psychological inflexibility [34]: the Psychology Inflexibility in Pain Scale (PIPS) has been developed to assess this core construct in ACT. It is a 16-item self-administered instrument, with two main factors labelled "avoidance" and "cognitive fusion". Participants are asked to rate how true these statements are on a 7-point Likert-type scale that ranges from ''never true'' to ''always true'', with higher scores indicating more psychological inflexibility. Its psychometric properties are considered adequate. We will validate a Spanish version of this test on an independent sample to be used in the study.

- Perceived injustice: a 12-item self-report measure, the Injustice Experience Questionnaire (IEQ) [19], was developed to measure this concept. It addresses the degree to which individuals perceive their post-disorder life as being characterized by injustice. Principal component analysis yielded a two-component solution. These factors were labelled "irreparability of loss" and "blame/unfairness". The psychometric properties of IEQ are considered adequate. We will validate a Spanish version of this test on an independent sample to be used in the study.

- Mindfulness: Several questionnaires have been used to assess the concept of mindfulness. For this study we will use one of the most widely used, the Mindful Attention Awareness Scale [21]. This is a 15-item self-administered questionnaire. Principal component analysis yielded a one-factor solution. The respondents indicate how frequently they have the experience described in each statement using a 6-point Likert scale from 1 (*almost always*) to 6 (*almost never*), where high scores reflect more mindfulness. In an attempt to monitor for socially-desirable responses, respondents are asked to answer according to what "really reflects" their experience rather than what they think their experience should be. The items are distributed across cognitive, emotional, physical, interpersonal and general domains. Psychometric properties of the questionnaire are adequate [21]. A Spanish version of this test will be validated on an independent sample to be used in the study.

-Positive and Negative Affect: This will be measured with the Positive and Negative Affect Scale (PANAS) [35]. The PANAS consists of 2 mood scales with 10 items each for the assessment of PA and NA. Scores for each scale range from 0 to 50. The scales have been shown to be internally consistent, uncorrelated and stable over a 2-month period; good convergent and discriminant validity have also been demonstrated [35]. The Spanish validated version will be used [36].

##### Other variables

- Psychiatric diagnosis: This will be performed with the MINI psychiatric interview [37], a psychiatric interview developed by our group and extensively used for the study of psychiatric disorders in primary care. It allows the use of DSM-IV diagnostic criteria. We specifically assess the presence of Post-traumatic Stress Disorders because it seems to be associated with a worse prognosis [38].

- Hospital Anxiety Depression Scale (HADS): This is a self-reported scale designed to screen for the presence of depression and anxiety disorders in medically ill patients. It contains 14 items rated on four-point Likert-type scales. Two subscales assess depression and anxiety independently (HADS-Dep and HADS-Anx, respectively) [39]. Patients with 14 or more points in the complete scale (or more than eight in either of the two subscales) are considered "probable cases" of anxiety and/or depression. The validated Spanish version of this questionnaire will be used [40].

### Procedure

Patients diagnosed with FM will be recruited by GPs from the selected health centres to fill the required sample. At baseline, trained researchers will administer questionnaires to assess socio-demographic, main outcome and other variables, and pain-related psychological constructs. At one-year follow-up, other researchers who will be blind to the results at baseline will administer the main outcome variables to the same patients.

### Statistical methods

#### Sample size

To calculate the sample size, it is necessary to know the population that suffers from FM in the region of Aragon. According to epidemiological studies [2] the prevalence of FM in Spain is 2-3%. The population of Aragon is calculated at 1,250,000 inhabitants, and based on this prevalence data the number of persons diagnosed with FM could be 30,000 patients. With a confidence level of 95%, an estimated error of 5% and considering the most unfavourable assumption (*p *= 0.5), a sample of 305 patients will be necessary, using an accuracy of 3%. EPIDAT 3.1 was used to calculate the sample size.

#### Analysis strategy

At baseline, means and standard deviations will be calculated for continuous variables in the study in case of normality, or medians and interquartile periods where this hypothesis is not fulfilled. The Kolmogorov-Smirnov test will be applied to ensure the adjustment of data to a normal distribution.

The existence of differences in socio-demographic, main outcome and other variables regarding pain-related psychological constructs will be analysed. These differences will be studied using Chi Square test for qualitative variables, with the Fisher test being utilized when necessary. To determine the relationship between categorical variables with one or two levels and quantitative variables, Student T test or variance analysis will be used, respectively, for variables fulfilling the normality hypothesis. For the remaining variables, non-parametric tests such as Man-Whitney U or Kruskall-Wallis will be used. To assess the predictive value of pain-related psychological construct on main outcome variables at one-year follow-up, use will be made of a logistic regression analysis adjusted for socio-demographic and clinical variables.

A Spearman Rho non-parametric correlation matrix will be developed to determine the relationship between pain-related psychological constructs and possible overlapping, including these constructs and the other variables, for the analysis of the significance of this matrix and each one of the calculated correlations. Patients will be grouped according to pain-related psychological constructs using cluster analysis, with a profile graph to represent the results of this multivariant analysis. Data will be analysed using the SPSS 15 statistical package.

### Ethical aspects

Informed consent will be obtained from the participants before commencing the study. Before they give their consent, patients will be provided with a general overview of the aims and characteristics of the study. They will also be informed that they will be participating voluntarily, and that they can choose to withdraw at any time with the guarantee that they will continue to receive the treatment considered most appropriate by their doctor. The study follows Helsinki Convention norms and subsequent modifications, and the Declaration of Madrid of the World Psychiatric Association. The Study Protocol was approved by the Ethical Review Board of the regional health authority (ref: PI09/041).

### Forecast execution dates

Initial recruitment of patients: December 2010

Finalization of patient recruitment: September 2011

Finalization of patient monitoring period: September 2012

Publication of results: December 2012

## Discussion

In recent years, the importance of cognitive and affective aspects for the treatment of chronic pain, not only in FM but also in other chronic pain diseases, has been widely acknowledged [41]. Furthermore, the effect of specific psychological constructs or pain-coping activities such as catastrophizing on the functioning of neuromuscular, immune and neuroendocrine systems is becoming an important subject in pain research [42]. Many of these constructs have been described and questionnaires for their assessment have been developed [43]. However, the importance of these constructs, the relationship and possible overlapping between them, or the exact meaning of them in pain are not enough known. For instance, pain acceptance seems to be a more general concept that includes acceptance of other kinds of unpleasant experiences, and it seems that general acceptance and not only pain acceptance could have positive effects on pain [44]. We consider this study thoroughly analyses the effect of a wide set of pain-related psychological constructs for the first time, allowing their effect on pain and function in chronic pain disorders to be understood.

Some possible limitations could be that recruitment, despite being randomized, may not include enough patients scoring positively in some of the psychological constructs, meaning that some of them cannot be studied. Another limitation is that, for some experts, the very concept of some of these constructs such as catastrophizing has been criticized, suggesting the need of new tools to assess them [45].

## Competing interests

The authors declare that they have no competing interests.

## Authors' contributions

JGC, JVL, BR and MR are the principal researchers and developed the original idea for the study. The study design was further developed by SM, YLH, RMB and YRL participated in the design and planning of the intervention that is evaluated here. EA developed the statistical methods. All authors have read and corrected draft versions, and approved the final version.

## Pre-publication history

The pre-publication history for this paper can be accessed here:

http://www.biomedcentral.com/1471-2474/12/4/prepub
